# Hepatitis D epidemiology and access to diagnostic testing among healthcare providers in Africa: A multi-country survey

**DOI:** 10.1016/j.jhepr.2025.101495

**Published:** 2025-07-03

**Authors:** Maria Buti, C. Wendy Spearman, Karin Siebelt, Manal El-Sayed

**Affiliations:** 1 Liver Unit, Internal Medicine Department, Hospital Universitari Vall d’Hebron, Vall d’Hebron Barcelona Hospital Campus, Barcelona, Spain; 2 Universitat Autònoma de Barcelona, Spain; 3 Centro de Investigación Biomédica en Red de Enfermedades Hepáticas y Digestivas (CIBERehd), Instituto de Salud Carlos III, Madrid, Spain; 4 Division of Hepatology, Department of Medicine, Faculty of Health Sciences, University of Cape Town, South Africa; 5 Academic Medical Education, Utrecht, The Netherlands; 6 Ain Shams University, Cairo, Egypt

**Keywords:** Hepatitis D, Epidemiology, Diagnosis, Survey

## Abstract

**Background & Aims:**

Reliable data on the prevalence of chronic HDV infection in Africa are limited. To address this, a multi-country survey was conducted across Africa to assess healthcare providers’ knowledge of HDV prevalence and the availability of diagnostic testing. This was complemented by a literature review of regional HDV prevalence data.

**Methods:**

A 12-item web-based questionnaire, created using Google forms, was distributed to all members of SOLDA (the Society on Liver Disease in Africa) and Project ECHO Viral Hepatitis in sub-Saharan Africa (n = 1,210) through African network channels. Survey responses were analyzed using descriptive statistics; all analyses were performed using GraphPad Prism 6.

**Results:**

A total of 1,210 surveys were distributed and completed by 608 participants across 43/54 (80%) African countries (44% Eastern, 36% Western, 8% Southern, 6% Northern, and 6% Central regions). Participants from 24/43 (56%) countries were aware of national HDV epidemiological data, mainly in relation to HBsAg carriers (77%), blood donors (23%), patients with chronic liver disease (25%), and those with hepatocellular carcinoma (18%). Anti-HDV antibody testing was available in 30/43 (69%) countries, primarily in clinical studies. The literature review identified 49 studies from 21 countries (mainly in Western and Central Africa), revealing a particularly high HDV prevalence in some countries (Cameroon, Gabon, and Nigeria). In 16 of 22 countries, survey participants’ awareness of HDV prevalence was consistent with published data.

**Conclusions:**

Healthcare providers’ knowledge of HDV prevalence varies across African countries, with 56% aware of national data and 73% aligned with published estimates. While diagnostic testing is available in 69% of countries, it remains limited, is seldom reimbursed, and is not routinely integrated into clinical practice.

**Impact and implications:**

This study provides the first continent-wide assessment of healthcare providers’ knowledge of HDV prevalence and diagnostic capacity across Africa. The findings reveal significant knowledge gaps – with nearly half of respondents unaware of national HDV data, particularly in Northern Africa – and limited availability of diagnostic testing in clinical practice. While anti-HDV testing is available in 69% of surveyed countries, it is often restricted to research settings, not reimbursed, and rarely integrated into routine care. A complementary literature review confirms that most published data originate from Western and Central Africa, with particularly high HDV prevalence reported in countries such as Cameroon, Gabon, and Nigeria. The study underscores the urgent need for improved HDV surveillance, provider education, and access to diagnostics. Strengthening these areas is essential to inform national hepatitis strategies, guide targeted interventions, and support WHO viral hepatitis elimination goals in the African region.

## Highlights

• This is one of the first African surveys on healthcare providers’ knowledge of HDV prevalence.
• Nearly half of respondents were unaware of HDV data, especially those from Northern Africa.
• Most published HDV studies were from Western Africa, followed by Central Africa.
• HDV prevalence was particularly elevated in Cameroon, Gabon, and Nigeria.
• Access to anti-HDV and HDV RNA testing is limited, seldom reimbursed, and not part of routine care.

## Graphical abstract

**Figure:**
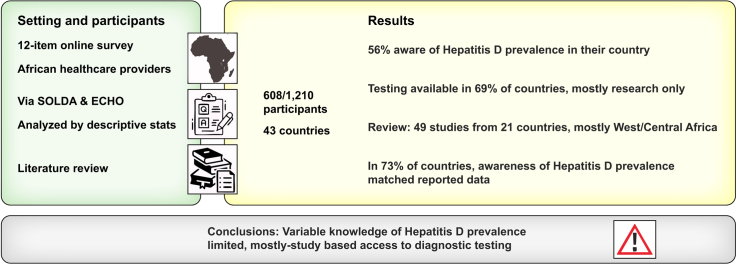

## Introduction

Chronic HDV infection is the most aggressive form of viral hepatitis. Patients with chronic HDV progress more rapidly to cirrhosis and hepatocellular carcinoma than those with chronic hepatitis B monoinfection.^1^ Globally, an estimated 4.5% of the HBsAg-positive population is coinfected with HDV, approximately 12 million people worldwide.^2^ HDV prevalence in the general population varies by region, from 3% in Europe to 6% in Africa. It is estimated that 1.6 million (95% CI 1.1–2.5) people are living with HDV in the World Health Organization (WHO) AFRO region.^2^^,^^3^ Hot spots of HDV infection have been reported in Mongolia, the Republic of Moldovia, and some countries in Western and Central Africa.^2^

A systematic review and meta-analysis of HDV prevalence in sub-Saharan Africa, published in 2017, yielded mixed epidemiological data. The pooled HDV seroprevalence in the HBsAg-positive population was 7.33% (95% CI 3.55%-12.20%) in Western Africa and 25.64% (12%-42%) in Central Africa. As would be expected, the prevalence was higher in populations with liver disease, reaching 9.57% (2.31%-20.43%) in Western Africa and 37.77% (12.13%-67.54%) in Central Africa. However, in Eastern and Southern Africa, the rates were relatively low, ranging from 0.05% to 1.78%.^3^ Overall, prevalence is higher in high-risk groups, such as people living with HIV, and increases with the severity of liver disease, particularly in patients with cirrhosis or hepatocellular carcinoma.[2], [3], [4], [5]

Accurate assessment of HDV prevalence is challenging for several reasons. First, large sample sizes of HBsAg-positive individuals are required to obtain consistent data. Second, testing for anti-HDV antibodies is not widely available in all countries, and HDV RNA testing to determine active infection may be based on non-standardized techniques such as in-house PCRs, making the diagnosis uncertain. Third, ongoing HBV vaccination programs have an impact in reducing HDV prevalence, particularly among children and adolescents. Therefore, studies are needed to update the data. The lack of access to accurate HDV diagnostics, limited up-to-date prevalence data, and low awareness of HDV among healthcare providers all contribute to HDV infection being under-recognized as a cause of liver disease-related morbidity and mortality.[1], [2], [3], [4], [5], [6]

The 2024 updated WHO Hepatitis B Management Guidelines recommend HDV reflex testing for all individuals who test positive for HBsAg.^7^ With the development of effective anti-HDV therapies, it is essential to determine the prevalence of this infection in the general population and specific high-risk groups in Africa to guide clinical care, establish policy measures, and inform effective public health interventions.[7], [8], [9]

This study aimed to assess healthcare professionals’ knowledge of HDV epidemiology and the availability of HDV screening and diagnostic tests in African countries, using a dedicated survey conducted across the continent. In addition, a literature review was carried out to collect existing data on HDV prevalence in Africa.

## Materials and methods

### Survey

A prospective, cross-sectional, web-based survey was designed by members of SOLDA (the Society on Liver Disease in Africa) and EASL (the European Association for the Study of the Liver), both organizations dedicated to the care of patients with liver diseases. The survey, created using Google Forms (Google LLC, Mountain View, CA, USA) included multiple-choice items and open-ended questions. It was available on the websites of SOLDA and Project ECHO Viral Hepatitis in sub-Saharan Africa from 19 April 2024 to 6 June 2024. All members of these societies (n = 1,210) were invited by email to voluntarily participate, with precautions taken to avoid duplicate invitations or responses from the same personnel at each center. The survey was also promoted on social media platforms, including the X and Facebook accounts of the participating societies.

The survey consisted of a 12-item questionnaire: one section for participants’ personal information and 11 questions related to the availability of HDV prevalence data and diagnostic tests, including their reimbursement status. Data were collected and categorized into five African regions: Northern, Eastern, Western, Central, and Southern Africa, depicted in the Figures.

### Literature review

A literature search was performed on PubMed, Embase, and Scopus, using the terms *hepatitis D*, *hepatitis delta*, *sub-Saharan Africa*, and the names of each African country. Eligible studies were those conducted in HBsAg-positive adults living in Africa, published in English as full papers between 1 January 1995 and 28 February 2025, and using anti-HDV antibody detection for the diagnosis. Reports included had to describe patient selection, methods for testing anti-HDV antibodies and HDV RNA, and the screening setting (community or clinical). We excluded studies published in other languages, those with small sample sizes (fewer than 25 participants) to avoid sampling bias, studies involving children, those that did not report the screening setting, and those using HDV antigen detection in serum or liver. Testing for HDV RNA was not an inclusion or exclusion criterion, but was taken into account when reported. The review was performed following the PRISMA recommendations.^10^

### Statistical analysis

The survey analysis was limited to responses from healthcare providers who completed at least 75% of the questions. Data collected from the survey were analyzed using descriptive statistics. Responses are summarized and presented in terms of frequencies and percentages to illustrate the distribution of answers. Anti-HDV prevalence is described among three groups: general populations, including blood donors and individuals tested in community surveys or antenatal clinics; hospital populations, comprising individuals tested in general hospitals and hepatology clinics, regardless of disease status; and selected population groups, such as people who inject drugs (PWID), individuals with HCV or HIV infection, and men who have sex with men. Categorical variables were compared using the chi-square test or the Fisher exact test when frequencies were less than 5%, and are expressed as frequency and percentage. Results were considered statistically significant at *p* values of less than 0.05. All statistical analyses were performed using GraphPad Prism 6. Confidence intervals were computed using the Wilson method.^11^

## Results

Surveys were completed by 608 healthcare providers from 43 of the 54 African countries (80%). Most respondents were from Eastern Africa (266 [44%]), followed by 217 (36%) from Western Africa, 50 (8%) from Southern Africa, 38 (6%) from Central Africa, and 37 (6%) from Northern Africa. No responses were received from invited participants in the following 11 countries: Angola, Cabo Verde, Chad, Comoros, Djibouti, Equatorial Guinea, Eritrea, Libya, Madagascar, São Tomé and Príncipe, and Seychelles.

### Knowledge of HDV prevalence

**Fig. 1:**
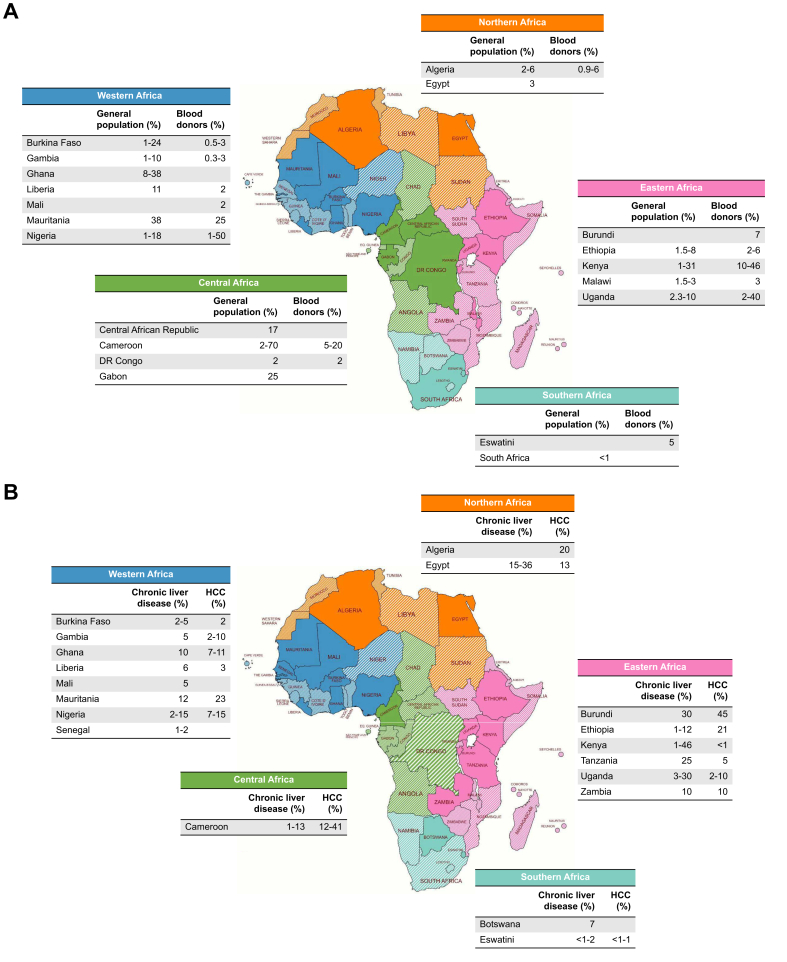
Survey responses regarding HDV prevalence across Africa. (A,B) Survey responses regarding HDV prevalence among HBsAg-positive cases in the general population and blood donors (A) and in patients with chronic liver disease and hepatocellular carcinoma (B) in five regions and various countries across Africa. Shaded colors indicate countries where prevalence was unknown or countries where no responses to the question were received.

Participants from 24 of the 43 African countries answering the survey (56%) were aware of HDV prevalence data in their setting (Fig. 1). By region, these included 4 of 5 (80%) countries in Central Africa, 8 of 14 (57%) in Western Africa, 7 of 13 (54%) in Eastern Africa, 3 of 5 (60%) in Southern Africa, and 2 of 6 (33%) in Northern Africa. The overall percentage of participants reporting knowledge of HDV prevalence data in specific populations was as follows: HBsAg carriers (77%), blood donors (23%), patients with chronic liver disease (25%), patients with hepatocellular carcinoma (18%), children (12%), adolescents 12%, dialysis patients (16%), and PWID (13%).

Data on HDV prevalence in HBsAg-positive blood donors and HBsAg carriers are presented together in Fig. 1A, while data in patients with chronic liver disease and hepatocellular carcinoma are shown in Fig. 1B. Additional information on the knowledge regarding HDV in other population groups (children, PWID, dialysis patients, etc.) is available in Table S1.

### Literature review

The literature search identified 49 publications and 5 reviews from Africa published between 2018 and 2025.[2], [3], [4], [5], [6]^,^[12], [13], [14], [15], [16], [17], [18], [19], [20], [21], [22], [23], [24], [25], [26], [27], [28], [29], [30], [31], [32], [33], [34], [35], [36], [37], [38], [39], [40], [41], [42], [43], [44], [45], [46], [47], [48], [49], [50], [51], [52], [53], [54], [55], [56], [57], [58], [59], [60] The prevalence of HDV infection reported by country, population, and type of study (community-based or hospital-based) is shown in Table 1. Most published studies were conducted in Western African populations (in total, 26) followed by 13 studies in Central Africa, 9 in Eastern Africa, 9 in Northern Africa, and 4 in Southern Africa. HDV prevalence was particularly elevated in certain countries in Central and Western Africa, such as Cameroon, Gabon, and Nigeria. HDV RNA was tested in 26 of the 61 populations included (43%) (Table 1). Diagnosis of active HDV infection was carried out in 21 of the 26 studies reporting HDV RNA testing, with detection ranging from 20% to 70.5%.

**Table 1:** HDV epidemiological results from the literature review of published studies.

Country	Author	Year	Sample size	Anti-HDV positive n (%)	95% CI	Sample size	HDV RNA detectable	95% CI
**General population**								
Burkina Faso^12^	Andernach IE	2014	40	1 (2.5%)	0-13	-	0	0-79
Cameroon^13^	Pinho-Nascimento CA	2018	54	8 (15%)	8-27	-	-	-
Cameroon^14^	Besombes C	2020	1,621	224 (11%)	10-13	-	-	-
Cameroon^15^	Noubissi-Jouegouo L	2019	426	70 (16%)	13-20		-	-
Cameroon^16^	Butler EK	2018	1,928	901 (47%)	45-49	887	624 (70.5%)	67-73
Central African Republic^17^	Komas NP	2018	110	5 (4.5%)	2-10	-	-	-
Egypt^18^	Gomaa NI	2013	170	8 (4.5%)	2-9	-	-	-
Ethiopia^19^	Tassachew Y	2023	323	25 (7.5%)	5-11	25	5 (20%)	9-39
Gabon^20^	Makuwa M	2009	124	82 (66%)	57-74	-	-	-
Gabon^21^	Groc S	2019	303	84 (28%)	23-33	-	-	-
Ghana^22^	Ampah KA	2016	107	9 (8%)	4-15	-	-	-
Nigeria^12^	Andernach IE	2014	326	40 (12%)	9-16	40	15 (37.5%)	24-53
Nigeria^23^	Sobajo OA	2023	51	2 (4%)	1-13	-	-	-
Nigeria^24^	Lemoine M	2016	394	3 (2%)	1-4	-	-	-
Tanzania^25^	Froeschl G	2021	64	1 (2%)	0-9	-	-	-
Tunisia^26^	Djebbi A	2009	176	12 (7%)	4-12	12	7 (54%)	28-77
Tunisia^27^	Mhalla S	2016	540	44 (8%)	6-11	-	-	-
Tunisia^28^	Triki H	1997	650	105 (16%)	13-19	-	-	-

**Blood donors**								
Burkina Faso^29^	Ouedraogo HG	2018	177	6 (3%)	1-7	-	-	-
Ethiopia^30^	Belyhun Y	2017	94	3 (3%)	1-9	3	1 (33.5%)	6-79
Mauritanie^31^	Mansour W	2012	447	90 (20%)	17-24	90	56 (62%)	52-72
Mozambique^32^	Cunha L	2007	146	0	0-3	-	-	-
Nigeria^24^	Lemoine M	2016	192	1 (<1%)	0-4	-	-	-

**Pregnant women**								
Benin^33^	De Paschale M	2014	44	5 (11%)	5-24	-	-	-
Burkina Faso^12^	Andernach IE	2014	49	0 (0%)	0-7	1	0	0-79
Cameroon^34^	Ndzie Ondigui JL	2024	130	42 (32%)	25-40	42	27 (62.5%)	47-76
Central African Republic^17^	Komas NP	2018	69	13 (19%)	11-30	-	-	-
Gabon^35^	Makuwa M	2008	109	17 (16%)	10-24	-	-	-
South Africa^36^	Andersson MI	2015	87	0	0-4	-	-	-

**HIV**								
Cameroon^37^	Torimiro JN	2018	64	40 (62.5%)	50-73	-	-	-
Ethiopia^30^	Belyhun Y	2017	125	10 (8%)	4-14	10	3 (30%)	11-60
Ghana^38^	Stockdale AJ	2018	222	5 (2%)	1-5	5	2 (40%)	12-77
Guinea-Bissau^39^	Hønge BL	2014	72	18 (25%)	16-36	9	4 (44%)	19-73
Malawi^38^	Stockdale AJ	2018	133	2 (1.5%)	0-5	2	0	0-71
Nigeria^12^	Andernach IE	2014	56	6 (11%)	5-22	6	2 (33%)	10-70
Nigeria^40^	Opayele OO	2021	50	36 (72%)	58-83	36	8 (22%)	12-38
Senagal^41^	Wembulua BS	2024	914	13 (1.5%)	1-2	13	8 (61.5%)	36-82
Senegal^42^	Diop-Ndiaye H	2008	61	2 (3%)	1-11	-	-	-
South Africa^36^	Andersson MI	2008	45	0	0-8	-	--	-
Tanzania^43^	Winter A	2016	219	11 (5%)	3-9	11	0	0-26
Uganda^44^	Katwesigye E	2017	198	6 (3%)	2-7	-	-	-

**Hospital population**								
Botswana^45^	Souda S	2021	153	7 (5%)	3-10	-	-	-
Egypt^46^	Makhlouf NA	2019	186	80 (43%)	36-50	80	25 (31%)	22-42
Egypt^47^	Elzefzafy W	2022	631	22 (3.5%)	2-5	22	8 (36.5)	20-57
Ethiopia^48^	Aberra H	2018	1,267	19 (1.5%)	1-2	19	12 (63)	41-81
Mauritanie^31^	Mansour W J	2012	162	31 (19%)	14-26	-	-	-
Nigeria^49^	Onyekwere CA	2012	245	5 (2%)	1-5	-	-	-
Nigeria^40^	Opaleye OO	2016	103	5 (5%)	2-11	5	0	0-43
Sudan^50^	Alajab MB	2024	90	8 (9%)	5-17	-	-	-

**Liver clinic**								
Algeria^51^	Gourari S	2019	112	6 (5%)	2-11	6	1 (16.5%)	3-56
Cameroon^52^	Luma HN	2017	294	31 (10.5%)	7-15	-	-	-
Cameroon^53^	Amougou MA	2016	58	24 (41%)	29-54	-	-	-
Ethiopia^30^	Belyhun Y	2017	102	13 (12.5%)	8-21	13	3 (23%)	8-50
Ghana^54^	Asmah RH	2014	53	6 (11%)	5-22	-	-	-
Mauritanie^55^	Lunel-Fabiani F	2013	296	98 (33%)	28-39	98	61 (67%)	57-76
Nigeria^12^	Andernach IE	2014	122	4 (3%)	1-8	4	1 (25%)	5-70
Nigeria^56^	Abdulkareem LO	2021	180	34 (19%)	14-25	-	-	-
Nigeria^57^	Nwokediuko SC	2009	96	12 (12.5%)	7-21	-	-	-
Nigeria^58^	Olal SO	2012	26	0	0-15		-	-
Senegal^59^	Vray M	2006	29	4 (14%)	6-31	-	-	-
Tunisia^60^	Yacoubi L	2015	1,615	33 (2%)	1-3	33	11 (33.5%)	20-50

In 16 of 22 countries, survey participants’ awareness of HDV prevalence was consistent with prevalence data reported in the literature. In contrast, participants from six countries reported being aware of data that was not available in the literature.

### Access to HDV diagnostic tests

**Fig. 2:**
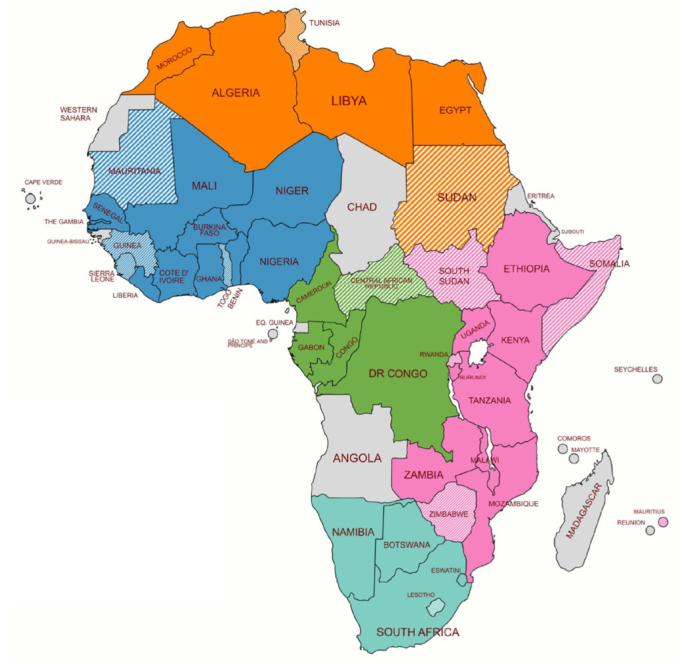
Countries with available anti-HDV testing according to the survey (n = 30). Shaded colors indicate countries without testing and grey indicates countries where no responses to this question were received.

**Fig. 3:**
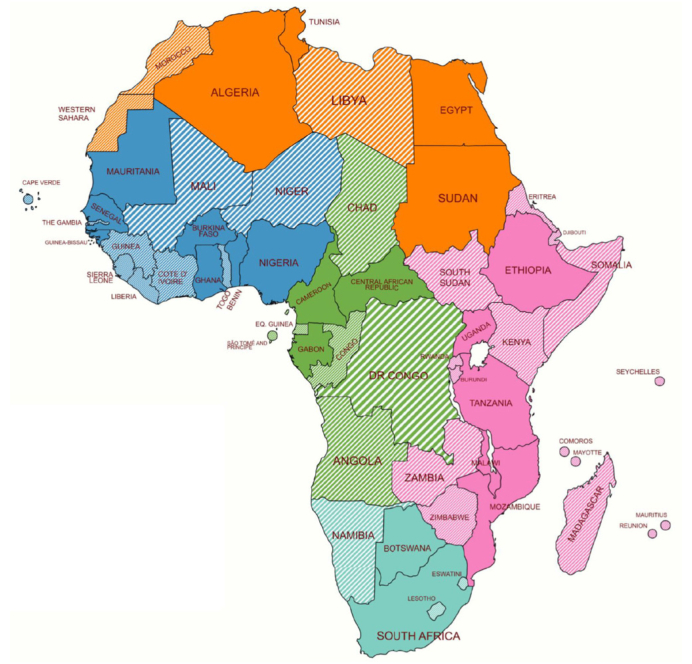
Countries with published anti-HDV data in the literature review (n = 21). Shaded colors indicate countries without published data.

According to the survey, participants from 30 of the 43 responding countries (69%) reported that anti-HDV antibody testing was available in their country (Fig. 2). However, responses within each country were mixed with both positive and negative answers reported. Overall, only 31% or fewer of all participants said the test was available to them. This suggests that two-thirds of participants did not have access to anti-HDV antibody testing, despite some national availability (Fig. 3). It is important to note that HDV RNA testing in all African countries is conducted in specific centers or for research studies, and is only reimbursed in a small percentage of cases (less than 10%). No African countries have integrated routine HDV testing into standard clinical practice.

The literature review showed that HDV RNA testing was performed in 13 out of 26 studies (50%) across various African countries. In all cases, HDV RNA was detected using an in-house PCR technique. No commercial tests were used.

## Discussion

This is the first survey conducted in Africa to assess healthcare providers’ knowledge regarding the prevalence of HDV infection in their respective regions. Despite several reports indicating a high prevalence of HDV infection in certain countries,[2], [3], [4], [5], [6] nearly 50% of participants in the survey were unaware of data on HDV prevalence in their region, particularly those living in Northern Africa.

A systematic review and meta-analysis from 2017 found substantial geographical variability in HDV prevalence in Africa. Regions such as Central and Western Africa, which had the majority of published studies, reported a relatively high HDV prevalence among both the general population and liver disease populations.^3^ This aligns with the results of our survey, which showed greater HDV awareness among healthcare workers from these regions.

We found that interpretation of HDV prevalence data from the survey results and the literature was challenging. In some cases, the findings in the literature were inconsistent, with studies reporting a higher HDV prevalence among HBsAg-positive carriers than among patients with chronic liver disease. Some of these studies involved small sample sizes, and most relied on detection of anti-HDV antibodies rather than HDV RNA for the diagnosis.^2^^,^^3^ Distinctions between HBsAg-positive chronic carriers and patients with liver disease were unclear in many studies, and some survey participants reported data for HBsAg-positive individuals without differentiating between chronic infection and chronic hepatitis. In addition, most studies and survey participants provided data on specific populations or locations, rather than regional or national data, further complicating interpretation of the results. This fragmented and incomplete epidemiological HDV data, with variable documentation in HBsAg carriers, children, blood donors, and patients with liver disease, hinders accurate assessment of HDV prevalence. Consequently, significant data gaps remain across Africa, emphasizing the need for more in-depth studies to obtain correct estimates of HDV epidemiology.

Another significant issue revealed by the survey is the limited access to HDV screening and diagnostic tests. In more than 60% of African countries with data, participants reported that anti-HDV antibody testing – the standard screening tool for HDV – is either unavailable or restricted to specific research studies. This, along with the limited implementation of international guidelines recommending anti-HDV testing for all HBsAg-positive individuals, has contributed to the underdiagnosis of HDV infection across Africa.^1^^,^^7^^,^[61], [62], [63] To illustrate this point, before the introduction of reflex anti-HDV testing in Spain, only 7.6% of HBsAg-positive patients were tested for anti-HDV,^64^ with similar findings reported in other countries.^65^ The use of reflex testing led to a five-fold increase in HDV diagnoses and enabled the detection of HBV/HDV coinfection in patients without known risk factors. In the Spanish study, around 60% of those diagnosed through reflex testing had no reported risk factors, underscoring the importance of testing all HBsAg-positive individuals.

Furthermore, access to HDV RNA testing is also limited in Africa. Survey participants noted that HDV RNA is mainly tested in research studies and, in some cases, is performed outside the country. Only half of the studies in the literature review reported HDV RNA results. A previous online global survey (208 respondents with 73 [35%] working in sub-Saharan Africa), conducted by ICE-HBV, assessed the availability of point-of-care HBV and HDV diagnostics. Only 2 of 73 respondents (3%) reported routine anti-HDV screening in HBsAg-positive patients, and only 9 (13%) had access to HDV RNA PCR testing.^66^ Our study provides additional evidence of the limited access to anti-HDV serological testing and HDV PCR in Africa.

One of the most pressing challenges revealed by the survey is the widespread lack of reimbursement for HDV screening and diagnostic tests. Only 10% of participants reported that HDV tests were reimbursed, and this was typically related to specific research projects. This lack of funding remains a significant barrier to understanding HDV epidemiology and disease burden in Africa, and the cost of diagnostics, which is mainly out-of-pocket, impedes an effective diagnostic pathway and linkage to care.^7^

The suboptimal awareness of HDV infection among healthcare providers, limited access to reliable serological screening tests, and the need for costly equipment and specialized training for confirmatory molecular HDV analysis, particularly in regions with inadequate medical infrastructure, has a detrimental impact on the diagnosis and proper care of HDV-infected individuals in Africa. However, the recent development of a rapid test for detecting anti-HDV antibodies in serum and plasma, with activity across all known HDV genotypes, may help address this situation.^67^ This novel anti-HDV test could become a valuable tool for both epidemiological studies and clinical diagnostics, particularly in regions lacking reliable HDV testing, as it covers all HDV genotypes including the African genotypes 5 to 8.

The strength of this study lies in its multi-center, multi-country design, which helps contextualize our findings within a broad African framework. However, there was considerable regional imbalance in the survey, with most respondents coming from Western and Eastern Africa, and fewer from Northern and Central Africa. Several African countries, including some with large populations, were not represented. Therefore, future studies should aim to include healthcare providers from the under-represented countries and regions. This study focused solely on healthcare personnel and assessed aspects strictly related to HDV prevalence awareness and access to diagnostic testing. Future surveys should also include the patients’ and policymakers’ viewpoints to evaluate key issues such as stigma and the reimbursement of diagnostic tests.

In conclusion, this survey on HDV infection in Africa found that awareness of HDV prevalence and access to diagnostic tests is limited and suboptimal, though there is substantial regional variability. Further research is needed to explore disease awareness, stigma, and patient perspectives, particularly in under-represented countries.

## Abbreviations

PWID, people who inject drugs; WHO, World Health Organization.

## Financial support

The authors did not receive any financial support to produce this manuscript.

## Authors’ contributions

Wendy Spearman, Karin Siebelt, Manal El-Sayed and Maria Buti contributed to the study concept and design, to the data acquisition and data analysis. All authors contributed to the analysis and interpretation of data, drafting of the manuscript and critical revision of the manuscript. All authors approved the final version of the manuscript.

## Data availability

The datasets generated and/or analyzed during the current study are available from the corresponding author upon reasonable request. All data supporting the findings of this study are included in the article and/or its supplementary materials.

## Conflict of interest

Wendy Spearman received honoraria for lectures from Gilead Sciences, Sanofi and Novodisk, Karin Siebelt and, Manal El-Sayed had not conflict of interest and Maria Buti received honoraria for lectures from Gilead Sciences, GSK and Vir.

Please refer to the accompanying ICMJE disclosure forms for further details.
