# Supplementary material for: Hepatitis D epidemiology and access to diagnostic testing among healthcare providers in Africa: A multi-country survey
Source: JHEP Rep. 2025 Jul 3;7(9):101495. doi: 10.1016/j.jhepr.2025.101495 (PMC12355062; doi:10.1016/j.jhepr.2025.101495)
Supplement: Multimedia component 1 [file mmc1.pdf]

Hepatitis D epidemiology and access to diagnostic testing among healthcare providers in Africa: A multi-country survey

Maria Buti, C. Wendy Spearman, Karin Siebelt, Manal El-Sayed

Table of content

Table S1……………………………………………………………………………2

Table S1: The table shows the percentage of participants who were aware of HDV data on the following populations: Children, Hemodialysis patients and People Who Injects Drugs (PWIDs).

North Africa	Children	Dialysis	PWIDs
Algeria	NA	11%	NA
Egypt	NA	NA	NA
Libya	NA	NA	NA
Morocco	NA	NA	NA
Sudan	NA	NA	NA
Tunisia	NA	NA	NA
West Africa	Children	Dialysis	PWIDs
Benin	NA	NA	NA
Burkina Faso	6-20,5%	NA	4%
Côte d'Ivoire	NA	NA	NA
The Gambia	5%	2%	4%
Ghana	NA	NA	10%
Guinea	NA	NA	NA
Liberia	3%	NA	1%
Mali	NA	NA	NA
Mauritania	14%	32%	NA
Niger	NA	NA	NA
Nigeria	5,5-11,4%	0-58%	0,1-40%
Senegal	NA	NA	NA
Sierra Leone	NA	NA	NA
Togo	NA	NA	NA
Central Africa	Children	Dialysis	PWIDs
Cameroon	1-50,5%	2%	NA
Central African Republic	16%	NA	NA
Congo-Kinshasa	NA	NA	NA
Democratic Republic of Congo	NA	NA	NA
Gabon	50%	NA	NA
East Africa	Children	Dialysis	PWIDs
Burundi	NA	15%	NA
Ethiopia	NA	NA	NA
Kenya	13%	24.3%	NA
Malawi	1,5-2%	NA	NA
Mauritius	NA	NA	NA
Mozambique	NA	NA	NA
Rwanda	NA	NA	NA
Somalia	NA	NA	NA
South Sudan	NA	NA	NA
Uganda	4-5%	1,5-45%	5-50%
Tanzania	3,3-60%	14%	20-27%
Zambia	1-5%	7%	10%
Zimbabwe	NA	NA	NA
Southern Africa	Children	Dialysis	PWIDs

**Botswana**	NA	NA	NA
**Eswatini**	NA	10%	NA
**Namibia**	NA	NA	NA
**Lesotho**	NA	NA	NA
**Namibia**	NA	NA	NA
**South Africa**	NA	NA	NA
