# Supplementary material for: Hepatitis D epidemiology and access to diagnostic testing among healthcare providers in Africa: A multi-country survey
Source: JHEP Rep. 2025 Jul 3;7(9):101495. doi: 10.1016/j.jhepr.2025.101495 (PMC12355062; doi:10.1016/j.jhepr.2025.101495)
Supplement: Multimedia component 4 [file mmc4.pdf]

JHEP|Reports

Hepatitis D epidemiology and access to diagnostic testing among healthcare providers in Africa: A multi-country survey

Authors

Maria Buti, C. Wendy Spearman, Karin Siebelt, Manal El-Sayed

Correspondence

mariabutiferret@gmail.com (M. Buti).

Graphical abstract

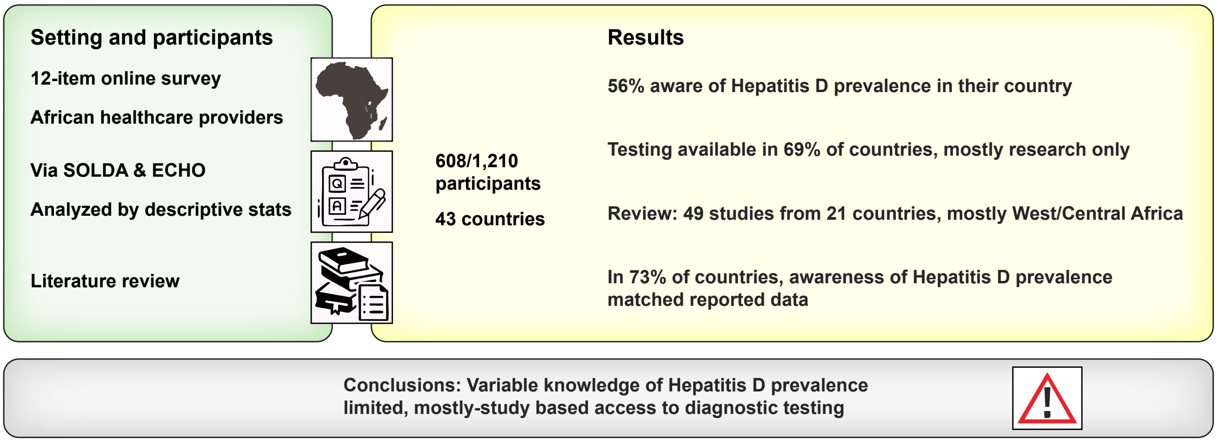

Highlights:

- This is one of the first African surveys on healthcare providers' knowledge of HDV prevalence.
- Nearly half of respondents were unaware of HDV data, especially those from Northern Africa.
- Most published HDV studies were from Western Africa, followed by Central Africa.
- HDV prevalence was particularly elevated in Cameroon, Gabon, and Nigeria.
- Access to anti-HDV and HDV RNA testing is limited, seldom reimbursed, and not part of routine care.

Impact and implications:

This study provides the first continent-wide assessment of healthcare providers' knowledge of HDV prevalence and diagnostic capacity across Africa. The findings reveal significant knowledge gaps – with nearly half of respondents unaware of national HDV data, particularly in Northern Africa – and limited availability of diagnostic testing in clinical practice. While anti-HDV testing is available in 69% of surveyed countries, it is often restricted to research settings, not reimbursed, and rarely integrated into routine care. A complementary literature review confirms that most published data originate from Western and Central Africa, with particularly high HDV prevalence reported in countries such as Cameroon, Gabon, and Nigeria. The study underscores the urgent need for improved HDV surveillance, provider education, and access to diagnostics. Strengthening these areas is essential to inform national hepatitis strategies, guide targeted interventions, and support WHO viral hepatitis elimination goals in the African region.

https://doi.org/10.1016/j.jhepr.2025.101495

© 2025 The Author(s). Published by Elsevier B.V. on behalf of European Association for the Study of the Liver (EASL). This is an open access article under the CC BY license (http://creativecommons.org/licenses/by/4.0/).

Hepatitis D epidemiology and access to diagnostic testing among healthcare providers in Africa: A multi-country survey

Maria Buti[1,2,3,*], C. Wendy Spearman[4], Karin Siebelt[5], Manal El-Sayed[6]

JHEP Reports **2025**. vol. 7 | 1–8

Check for updates

Background & Aims: Reliable data on the prevalence of chronic HDV infection in Africa are limited. To address this, a multi-country survey was conducted across Africa to assess healthcare providers' knowledge of HDV prevalence and the availability of diagnostic testing. This was complemented by a literature review of regional HDV prevalence data.

Methods: A 12-item web-based questionnaire, created using Google forms, was distributed to all members of SOLDA (the Society on Liver Disease in Africa) and Project ECHO Viral Hepatitis in sub-Saharan Africa (n = 1,210) through African network channels. Survey responses were analyzed using descriptive statistics; all analyses were performed using GraphPad Prism 6.

Results: A total of 1,210 surveys were distributed and completed by 608 participants across 43/54 (80%) African countries (44% Eastern, 36% Western, 8% Southern, 6% Northern, and 6% Central regions). Participants from 24/43 (56%) countries were aware of national HDV epidemiological data, mainly in relation to HBsAg carriers (77%), blood donors (23%), patients with chronic liver disease (25%), and those with hepatocellular carcinoma (18%). Anti-HDV antibody testing was available in 30/43 (69%) countries, primarily in clinical studies. The literature review identified 49 studies from 21 countries (mainly in Western and Central Africa), revealing a particularly high HDV prevalence in some countries (Cameroon, Gabon, and Nigeria). In 16 of 22 countries, survey participants' awareness of HDV prevalence was consistent with published data.

Conclusions: Healthcare providers' knowledge of HDV prevalence varies across African countries, with 56% aware of national data and 73% aligned with published estimates. While diagnostic testing is available in 69% of countries, it remains limited, is seldom reimbursed, and is not routinely integrated into clinical practice.

© 2025 The Author(s). Published by Elsevier B.V. on behalf of European Association for the Study of the Liver (EASL). This is an open access article under the CC BY license (http://creativecommons.org/licenses/by/4.0/).

Introduction

Chronic HDV infection is the most aggressive form of viral hepatitis. Patients with chronic HDV progress more rapidly to cirrhosis and hepatocellular carcinoma than those with chronic hepatitis B monoinfection.[1] Globally, an estimated 4.5% of the HBsAg-positive population is coinfected with HDV, approximately 12 million people worldwide.[2] HDV prevalence in the general population varies by region, from 3% in Europe to 6% in Africa. It is estimated that 1.6 million (95% CI 1.1–2.5) people are living with HDV in the World Health Organization (WHO) AFRO region.[2,3] Hot spots of HDV infection have been reported in Mongolia, the Republic of Moldova, and some countries in Western and Central Africa.[2]

A systematic review and meta-analysis of HDV prevalence in sub-Saharan Africa, published in 2017, yielded mixed epidemiological data. The pooled HDV seroprevalence in the HBsAg-positive population was 7.33% (95% CI 3.55%-12.20%) in Western Africa and 25.64% (12%-42%) in Central Africa. As would be expected, the prevalence was higher in populations with liver disease, reaching 9.57% (2.31%-20.43%) in Western Africa and 37.77% (12.13%-67.54%) in Central Africa. However, in Eastern and Southern Africa, the rates were relatively low, ranging from 0.05% to 1.78%.[3] Overall, prevalence is higher in high-risk groups, such as people living with HIV, and increases with the severity of liver disease, particularly in patients with cirrhosis or hepatocellular carcinoma.[2–5]

Accurate assessment of HDV prevalence is challenging for several reasons. First, large sample sizes of HBsAg-positive individuals are required to obtain consistent data. Second, testing for anti-HDV antibodies is not widely available in all countries, and HDV RNA testing to determine active infection may be based on non-standardized techniques such as in-house PCRs, making the diagnosis uncertain. Third, ongoing HBV vaccination programs have an impact in reducing HDV prevalence, particularly among children and adolescents. Therefore, studies are needed to update the data. The lack of access to accurate HDV diagnostics, limited up-to-date prevalence data, and low awareness of HDV among healthcare providers all contribute to HDV infection being under-recognized as a cause of liver disease-related morbidity and mortality.[1–6]

The 2024 updated WHO Hepatitis B Management Guidelines recommend HDV reflex testing for all individuals

* Corresponding author. Address: Hospital Universitari Vall d'Hebron, Paseo Valle Hebrón 119-129; Barcelona 08035, Barcelona, Spain.
E-mail address: mariabutiferret@gmail.com (M. Buti).
https://doi.org/10.1016/j.jhepr.2025.101495

who test positive for HBsAg.[7] With the development of effective anti-HDV therapies, it is essential to determine the prevalence of this infection in the general population and specific high-risk groups in Africa to guide clinical care, establish policy measures, and inform effective public health interventions.[7–9]

This study aimed to assess healthcare professionals' knowledge of HDV epidemiology and the availability of HDV screening and diagnostic tests in African countries, using a dedicated survey conducted across the continent. In addition, a literature review was carried out to collect existing data on HDV prevalence in Africa.

Materials and methods

Survey

A prospective, cross-sectional, web-based survey was designed by members of SOLDA (the Society on Liver Disease in Africa) and EASL (the European Association for the Study of the Liver), both organizations dedicated to the care of patients with liver diseases. The survey, created using Google Forms (Google LLC, Mountain View, CA, USA) included multiple-choice items and open-ended questions. It was available on the websites of SOLDA and Project ECHO Viral Hepatitis in sub-Saharan Africa from 19 April 2024 to 6 June 2024. All members of these societies (n = 1,210) were invited by email to voluntarily participate, with precautions taken to avoid duplicate invitations or responses from the same personnel at each center. The survey was also promoted on social media platforms, including the X and Facebook accounts of the participating societies.

The survey consisted of a 12-item questionnaire: one section for participants' personal information and 11 questions related to the availability of HDV prevalence data and diagnostic tests, including their reimbursement status. Data were collected and categorized into five African regions: Northern, Eastern, Western, Central, and Southern Africa, depicted in the Figures.

Literature review

A literature search was performed on PubMed, Embase, and Scopus, using the terms *hepatitis D*, *hepatitis delta*, *sub-Saharan Africa*, and the names of each African country. Eligible studies were those conducted in HBsAg-positive adults living in Africa, published in English as full papers between 1 January 1995 and 28 February 2025, and using anti-HDV antibody detection for the diagnosis. Reports included had to describe patient selection, methods for testing anti-HDV antibodies and HDV RNA, and the screening setting (community or clinical). We excluded studies published in other languages, those with small sample sizes (fewer than 25 participants) to avoid sampling bias, studies involving children, those that did not report the screening setting, and those using HDV antigen detection in serum or liver. Testing for HDV RNA was not an inclusion or exclusion criterion, but was taken into account when reported. The review was performed following the PRISMA recommendations.[10]

Statistical analysis

The survey analysis was limited to responses from healthcare providers who completed at least 75% of the questions. Data collected from the survey were analyzed using descriptive statistics. Responses are summarized and presented in terms of frequencies and percentages to illustrate the distribution of answers. Anti-HDV prevalence is described among three groups: general populations, including blood donors and individuals tested in community surveys or antenatal clinics; hospital populations, comprising individuals tested in general hospitals and hepatology clinics, regardless of disease status; and selected population groups, such as people who inject drugs (PWID), individuals with HCV or HIV infection, and men who have sex with men. Categorical variables were compared using the chi-square test or the Fisher exact test when frequencies were less than 5%, and are expressed as frequency and percentage. Results were considered statistically significant at p values of less than 0.05. All statistical analyses were performed using GraphPad Prism 6. Confidence intervals were computed using the Wilson method.[11]

Results

Surveys were completed by 608 healthcare providers from 43 of the 54 African countries (80%). Most respondents were from Eastern Africa (266 [44%]), followed by 217 (36%) from Western Africa, 50 (8%) from Southern Africa, 38 (6%) from Central Africa, and 37 (6%) from Northern Africa. No responses were received from invited participants in the following 11 countries: Angola, Cabo Verde, Chad, Comoros, Djibouti, Equatorial Guinea, Eritrea, Libya, Madagascar, São Tomé and Príncipe, and Seychelles.

Knowledge of HDV prevalence

Participants from 24 of the 43 African countries answering the survey (56%) were aware of HDV prevalence data in their setting (Fig. 1). By region, these included 4 of 5 (80%) countries in Central Africa, 8 of 14 (57%) in Western Africa, 7 of 13 (54%) in Eastern Africa, 3 of 5 (60%) in Southern Africa, and 2 of 6 (33%) in Northern Africa. The overall percentage of participants reporting knowledge of HDV prevalence data in specific populations was as follows: HBsAg carriers (77%), blood donors (23%), patients with chronic liver disease (25%), patients with hepatocellular carcinoma (18%), children (12%), adolescents 12%, dialysis patients (16%), and PWID (13%).

Data on HDV prevalence in HBsAg-positive blood donors and HBsAg carriers are presented together in Fig. 1A, while data in patients with chronic liver disease and hepatocellular carcinoma are shown in Fig. 1B. Additional information on the knowledge regarding HDV in other population groups (children, PWID, dialysis patients, etc.) is available in Table S1.

Literature review

The literature search identified 49 publications and 5 reviews from Africa published between 2018 and 2025.[2–6,12–60] The prevalence of HDV infection reported by country, population, and type of study (community-based or hospital-based) is shown in Table 1. Most published studies were conducted in Western African populations (in total, 26) followed by 13 studies in Central Africa, 9 in Eastern Africa, 9 in Northern Africa, and 4 in Southern Africa. HDV prevalence was particularly elevated in certain countries in Central and Western Africa, such as Cameroon, Gabon, and Nigeria. HDV RNA was

A

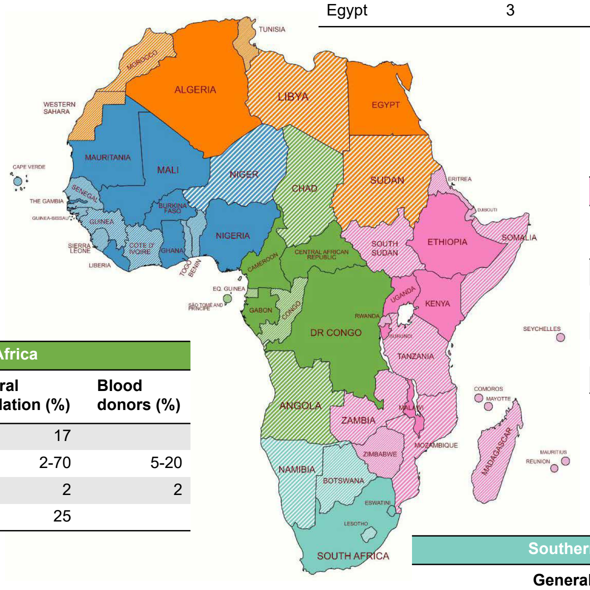

Northern Africa	General population (%)	Blood donors (%)
Algeria	2-6	0.9-6
Egypt	3	

Western Africa	General population (%)	Blood donors (%)
Burkina Faso	1-24	0.5-3
Gambia	1-10	0.3-3
Ghana	8-38	
Liberia	11	2
Mali		2
Mauritania	38	25
Nigeria	1-18	1-50

Eastern Africa	General population (%)	Blood donors (%)
Burundi		7
Ethiopia	1.5-8	2-6
Kenya	1-31	10-46
Malawi	1.5-3	3
Uganda	2.3-10	2-40

Central Africa	General population (%)	Blood donors (%)
Central African Republic	17	
Cameroon	2-70	5-20
DR Congo	2	2
Gabon	25	

Southern Africa	General population (%)	Blood donors (%)
Eswatini		5
South Africa	<1	

B

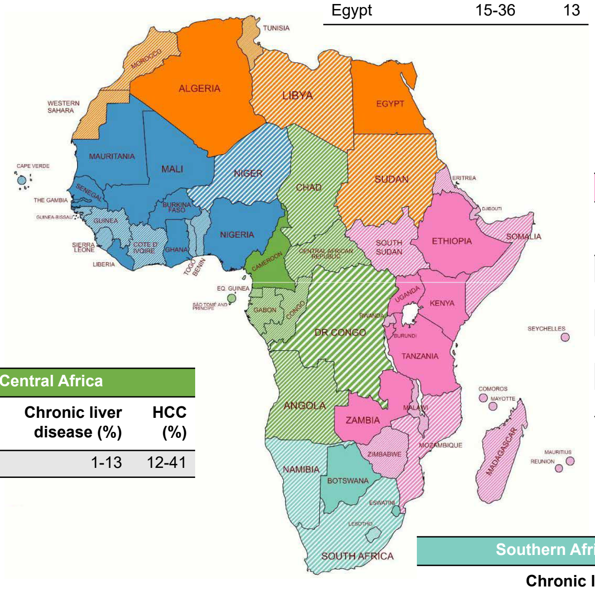

Northern Africa	Chronic liver disease (%)	HCC (%)
Algeria		20
Egypt	15-36	13

Western Africa	Chronic liver disease (%)	HCC (%)
Burkina Faso	2-5	2
Gambia	5	2-10
Ghana	10	7-11
Liberia	6	3
Mali	5	
Mauritania	12	23
Nigeria	2-15	7-15
Senegal	1-2	

Eastern Africa	Chronic liver disease (%)	HCC (%)
Burundi	30	45
Ethiopia	1-12	21
Kenya	1-46	<1
Tanzania	25	5
Uganda	3-30	2-10
Zambia	10	10

Central Africa	Chronic liver disease (%)	HCC (%)
Cameroon	1-13	12-41

Southern Africa	Chronic liver disease (%)	HCC (%)
Botswana	7	
Eswatini	<1-2	<1-1

Fig. 1. Survey responses regarding HDV prevalence across Africa. (A,B) Survey responses regarding HDV prevalence among HBsAg-positive cases in the general population and blood donors (A) and in patients with chronic liver disease and hepatocellular carcinoma (B) in five regions and various countries across Africa. Shaded colors indicate countries where prevalence was unknown or countries where no responses to the question were received.

Table 1. HDV epidemiological results from the literature review of published studies.

Country	Author	Year	Sample size	Anti-HDV positive n (%)	95% CI	Sample size	HDV RNA detectable	95% CI
**General population**								
Burkina Faso[12]	Andernach IE	2014	40	1 (2.5%)	0-13	-	0	0-79
Cameroon[13]	Pinho-Nascimento CA	2018	54	8 (15%)	8-27	-	-	-
Cameroon[14]	Besombes C	2020	1,621	224 (11%)	10-13	-	-	-
Cameroon[15]	Noubissi-Jouegouo L	2019	426	70 (16%)	13-20		-	-
Cameroon[16]	Butler EK	2018	1,928	901 (47%)	45-49	887	624 (70.5%)	67-73
Central African Republic[17]	Komas NP	2018	110	5 (4.5%)	2-10	-	-	-
Egypt[18]	Gomaa NI	2013	170	8 (4.5%)	2-9	-	-	-
Ethiopia[19]	Tassachew Y	2023	323	25 (7.5%)	5-11	25	5 (20%)	9-39
Gabon[20]	Makuwa M	2009	124	82 (66%)	57-74	-	-	-
Gabon[21]	Groc S	2019	303	84 (28%)	23-33	-	-	-
Ghana[22]	Ampah KA	2016	107	9 (8%)	4-15	-	-	-
Nigeria[12]	Andernach IE	2014	326	40 (12%)	9-16	40	15 (37.5%)	24-53
Nigeria[23]	Sobajo OA	2023	51	2 (4%)	1-13	-	-	-
Nigeria[24]	Lemoine M	2016	394	3 (2%)	1-4	-	-	-
Tanzania[25]	Froeschl G	2021	64	1 (2%)	0-9	-	-	-
Tunisia[26]	Djebbi A	2009	176	12 (7%)	4-12	12	7 (54%)	28-77
Tunisia[27]	Mhalla S	2016	540	44 (8%)	6-11	-	-	-
Tunisia[28]	Triki H	1997	650	105 (16%)	13-19	-	-	-
**Blood donors**								
Burkina Faso[29]	Ouedraogo HG	2018	177	6 (3%)	1-7	-	-	-
Ethiopia[30]	Belyhun Y	2017	94	3 (3%)	1-9	3	1 (33.5%)	6-79
Mauritanie[31]	Mansour W	2012	447	90 (20%)	17-24	90	56 (62%)	52-72
Mozambique[32]	Cunha L	2007	146	0	0-3	-	-	-
Nigeria[24]	Lemoine M	2016	192	1 (<1%)	0-4	-	-	-
**Pregnant women**								
Benin[33]	De Paschale M	2014	44	5 (11%)	5-24	-	-	-
Burkina Faso[12]	Andernach IE	2014	49	0 (0%)	0-7	1	0	0-79
Cameroon[34]	Ndzie Ondigui JL	2024	130	42 (32%)	25-40	42	27 (62.5%)	47-76
Central African Republic[17]	Komas NP	2018	69	13 (19%)	11-30	-	-	-
Gabon[35]	Makuwa M	2008	109	17 (16%)	10-24	-	-	-
South Africa[36]	Andersson MI	2015	87	0	0-4	-	-	-
**HIV**								
Cameroon[37]	Torimiro JN	2018	64	40 (62.5%)	50-73	-	-	-
Ethiopia[30]	Belyhun Y	2017	125	10 (8%)	4-14	10	3 (30%)	11-60
Ghana[38]	Stockdale AJ	2018	222	5 (2%)	1-5	5	2 (40%)	12-77
Guinea-Bissau[39]	Hønge BL	2014	72	18 (25%)	16-36	9	4 (44%)	19-73
Malawi[38]	Stockdale AJ	2018	133	2 (1.5%)	0-5	2	0	0-71
Nigeria[12]	Andernach IE	2014	56	6 (11%)	5-22	6	2 (33%)	10-70
Nigeria[40]	Opayele OO	2021	50	36 (72%)	58-83	36	8 (22%)	12-38
Senagal[41]	Wembulua BS	2024	914	13 (1.5%)	1-2	13	8 (61.5%)	36-82
Senegal[42]	Diop-Ndiaye H	2008	61	2 (3%)	1-11	-	-	-
South Africa[36]	Andersson MI	2008	45	0	0-8	-	–	-
Tanzania[43]	Winter A	2016	219	11 (5%)	3-9	11	0	0-26
Uganda[44]	Katwesigye E	2017	198	6 (3%)	2-7	-	-	-
**Hospital population**								
Botswana[45]	Souda S	2021	153	7 (5%)	3-10	-	-	-
Egypt[46]	Makhlouf NA	2019	186	80 (43%)	36-50	80	25 (31%)	22-42
Egypt[47]	Elzefzafy W	2022	631	22 (3.5%)	2-5	22	8 (36.5)	20-57
Ethiopia[48]	Aberra H	2018	1,267	19 (1.5%)	1-2	19	12 (63)	41-81

(continued on next page)

Table 1. (*continued*)

Country	Author	Year	Sample size	Anti-HDV positive n (%)	95% CI	Sample size	HDV RNA detectable	95% CI
Mauritanie[31]	Mansour W J	2012	162	31 (19%)	14-26	-	-	-
Nigeria[49]	Onyekwere CA	2012	245	5 (2%)	1-5	-	-	-
Nigeria[40]	Opaleye OO	2016	103	5 (5%)	2-11	5	0	0-43
Sudan[50]	Alajab MB	2024	90	8 (9%)	5-17	-	-	-
**Liver clinic**								
Algeria[51]	Gourari S	2019	112	6 (5%)	2-11	6	1 (16.5%)	3-56
Cameroon[52]	Luma HN	2017	294	31 (10.5%)	7-15	-	-	-
Cameroon[53]	Amougou MA	2016	58	24 (41%)	29-54	-	-	-
Ethiopia[30]	Belyhun Y	2017	102	13 (12.5%)	8-21	13	3 (23%)	8-50
Ghana[54]	Asmah RH	2014	53	6 (11%)	5-22	-	-	-
Mauritanie[55]	Lunel-Fabiani F	2013	296	98 (33%)	28-39	98	61 (67%)	57-76
Nigeria[12]	Andernach IE	2014	122	4 (3%)	1-8	4	1 (25%)	5-70
Nigeria[56]	Abdulkareem LO	2021	180	34 (19%)	14-25	-	-	-
Nigeria[57]	Nwokediuko SC	2009	96	12 (12.5%)	7-21	-	-	-
Nigeria[58]	Olal SO	2012	26	0	0-15		-	-
Senegal[59]	Vray M	2006	29	4 (14%)	6-31	-	-	-
Tunisia[60]	Yacoubi L	2015	1,615	33 (2%)	1-3	33	11 (33.5%)	20-50

tested in 26 of the 61 populations included (43%) (Table 1). Diagnosis of active HDV infection was carried out in 21 of the 26 studies reporting HDV RNA testing, with detection ranging from 20% to 70.5%.

In 16 of 22 countries, survey participants' awareness of HDV prevalence was consistent with prevalence data reported in the literature. In contrast, participants from six countries reported being aware of data that was not available in the literature.

Access to HDV diagnostic tests

According to the survey, participants from 30 of the 43 responding countries (69%) reported that anti-HDV antibody testing was available in their country (Fig. 2). However, responses within each country were mixed with both positive and negative answers reported. Overall, only 31% or fewer of all participants said the test was available to them. This suggests that two-thirds of participants did not have access to anti-HDV antibody testing, despite some national availability (Fig. 3). It is important to note that HDV RNA testing in all African countries is conducted in specific centers or for research studies, and is only reimbursed in a small percentage of cases (less than 10%). No African countries have integrated routine HDV testing into standard clinical practice.

The literature review showed that HDV RNA testing was performed in 13 out of 26 studies (50%) across various African countries. In all cases, HDV RNA was detected using an in-house PCR technique. No commercial tests were used.

Discussion

This is the first survey conducted in Africa to assess healthcare providers' knowledge regarding the prevalence of HDV infection in their respective regions. Despite several reports

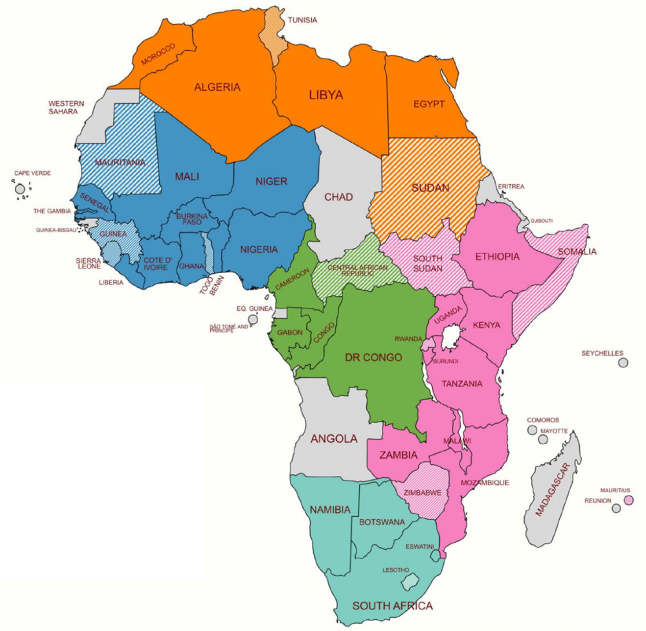

Fig. 2. Countries with available anti-HDV testing according to the survey (n = 30). Shaded colors indicate countries without testing and grey indicates countries where no responses to this question were received.

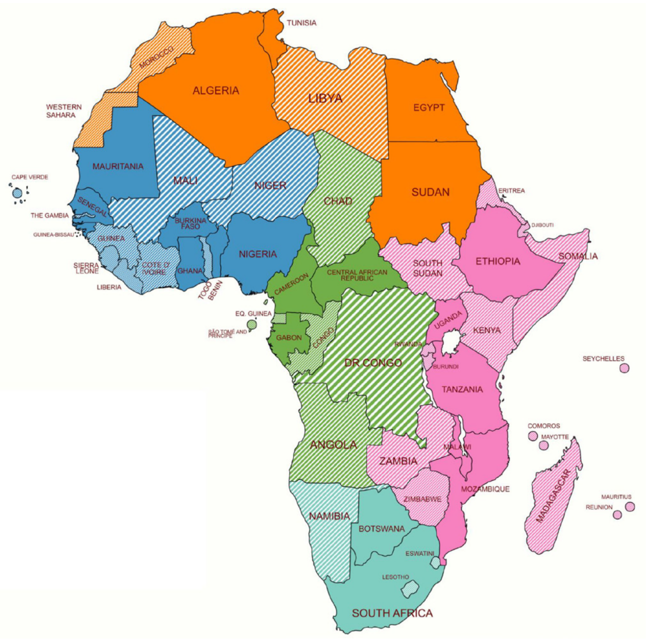

Fig. 3. Countries with published anti-HDV data in the literature review (n = 21). Shaded colors indicate countries without published data.

indicating a high prevalence of HDV infection in certain countries,[2–6] nearly 50% of participants in the survey were unaware of data on HDV prevalence in their region, particularly those living in Northern Africa.

A systematic review and meta-analysis from 2017 found substantial geographical variability in HDV prevalence in Africa. Regions such as Central and Western Africa, which had the majority of published studies, reported a relatively high HDV prevalence among both the general population and liver disease populations.[3] This aligns with the results of our survey, which showed greater HDV awareness among healthcare workers from these regions.

We found that interpretation of HDV prevalence data from the survey results and the literature was challenging. In some cases, the findings in the literature were inconsistent, with studies reporting a higher HDV prevalence among HBsAg-positive carriers than among patients with chronic liver disease. Some of these studies involved small sample sizes, and most relied on detection of anti-HDV antibodies rather than HDV RNA for the diagnosis.[2,3] Distinctions between HBsAg-positive chronic carriers and patients with liver disease were unclear in many studies, and some survey participants reported data for HBsAg-positive individuals without differentiating between chronic infection and chronic hepatitis. In addition, most studies and survey participants provided data on specific populations or locations, rather than regional or national data, further complicating interpretation of the results. This fragmented and incomplete epidemiological HDV data, with variable documentation in HBsAg carriers, children, blood donors, and patients with liver disease, hinders accurate assessment of HDV prevalence. Consequently, significant data gaps remain across Africa, emphasizing the need for more in-depth studies to obtain correct estimates of HDV epidemiology.

Another significant issue revealed by the survey is the limited access to HDV screening and diagnostic tests. In more than 60% of African countries with data, participants reported that anti-HDV antibody testing – the standard screening tool for HDV – is either unavailable or restricted to specific research studies. This, along with the limited implementation of international guidelines recommending anti-HDV testing for all HBsAg-positive individuals, has contributed to the underdiagnosis of HDV infection across Africa.[1,7,61–63] To illustrate this point, before the introduction of reflex anti-HDV testing in Spain, only 7.6% of HBsAg-positive patients were tested for anti-HDV,[64] with similar findings reported in other countries.[65] The use of reflex testing led to a five-fold increase in HDV diagnoses and enabled the detection of HBV/HDV coinfection in patients without known risk factors. In the Spanish study, around 60% of those diagnosed through reflex testing had no reported risk factors, underscoring the importance of testing all HBsAg-positive individuals.

Furthermore, access to HDV RNA testing is also limited in Africa. Survey participants noted that HDV RNA is mainly tested in research studies and, in some cases, is performed outside the country. Only half of the studies in the literature review reported HDV RNA results. A previous online global survey (208 respondents with 73 [35%] working in sub-Saharan Africa), conducted by ICE-HBV, assessed the availability of point-of-care HBV and HDV diagnostics. Only 2 of 73 respondents (3%) reported routine anti-HDV screening in HBsAg-positive patients, and only 9 (13%) had access to HDV RNA PCR testing.[66] Our study provides additional evidence of the limited access to anti-HDV serological testing and HDV PCR in Africa.

One of the most pressing challenges revealed by the survey is the widespread lack of reimbursement for HDV screening and diagnostic tests. Only 10% of participants reported that HDV tests were reimbursed, and this was typically related to specific research projects. This lack of funding remains a significant barrier to understanding HDV epidemiology and disease burden in Africa, and the cost of diagnostics, which is mainly out-of-pocket, impedes an effective diagnostic pathway and linkage to care.[7]

The suboptimal awareness of HDV infection among healthcare providers, limited access to reliable serological screening tests, and the need for costly equipment and specialized training for confirmatory molecular HDV analysis, particularly in regions with inadequate medical infrastructure, has a detrimental impact on the diagnosis and proper care of HDV-infected individuals in Africa. However, the recent development of a rapid test for detecting anti-HDV antibodies in serum and plasma, with activity across all known HDV genotypes, may help address this situation.[67] This novel anti-HDV test could become a valuable tool for both epidemiological studies and clinical diagnostics, particularly in regions lacking reliable HDV testing, as it covers all HDV genotypes including the African genotypes 5 to 8.

The strength of this study lies in its multi-center, multi-country design, which helps contextualize our findings within a broad African framework. However, there was considerable regional imbalance in the survey, with most respondents coming from Western and Eastern Africa, and fewer from Northern and Central Africa. Several African countries, including some with large populations, were not represented. Therefore, future

studies should aim to include healthcare providers from the under-represented countries and regions. This study focused solely on healthcare personnel and assessed aspects strictly related to HDV prevalence awareness and access to diagnostic testing. Future surveys should also include the patients' and policymakers' viewpoints to evaluate key issues such as stigma and the reimbursement of diagnostic tests.

In conclusion, this survey on HDV infection in Africa found that awareness of HDV prevalence and access to diagnostic tests is limited and suboptimal, though there is substantial regional variability. Further research is needed to explore disease awareness, stigma, and patient perspectives, particularly in under-represented countries.

Affiliations

[1]*Liver Unit, Internal Medicine Department, Hospital Universitari Vall d'Hebron, Vall d'Hebron Barcelona Hospital Campus, Barcelona, Spain;* [2]*Universitat Autònoma de Barcelona, Spain;* [3]*Centro de Investigación Biomédica en Red de Enfermedades Hepáticas y Digestivas (CIBERehd), Instituto de Salud Carlos III, Madrid, Spain;* [4]*Division of Hepatology, Department of Medicine, Faculty of Health Sciences, University of Cape Town, South Africa;* [5]*Academic Medical Education, Utrecht, The Netherlands;* [6]*Ain Shams University, Cairo, Egypt*

Abbreviations

PWID, people who inject drugs; WHO, World Health Organization.

Financial support

The authors did not receive any financial support to produce this manuscript.

Conflict of interest

Wendy Spearman received honoraria for lectures from Gilead Sciences, Sanofi and Novodisk, Karin Siebelt and, Manal El-Sayed had not conflict of interest and Maria Buti received honoraria for lectures from Gilead Sciences, GSK and Vir.

Please refer to the accompanying ICMJE disclosure forms for further details.

Authors' contributions

Wendy Spearman, Karin Siebelt, Manal El-Sayed and Maria Buti contributed to the study concept and design, to the data acquisition and data analysis. All authors contributed to the analysis and interpretation of data, drafting of the manuscript and critical revision of the manuscript. All authors approved the final version of the manuscript.

Data availability

The datasets generated and/or analyzed during the current study are available from the corresponding author upon reasonable request. All data supporting the findings of this study are included in the article and/or its supplementary materials.

Supplementary data

Supplementary data to this article can be found online at https://doi.org/10.1016/j.jhepr.2025.101495.

References

Author names in bold designate shared co-first authorship

[1] European Association for the Study of the Liver. EASL Clinical Practice Guidelines on hepatitis delta virus. J Hepatol 2023;79:433–460.
[2] Stockdale AJ, Kreuels B, Henrion MYR, et al. The global prevalence of hepatitis D virus infection: systematic review and meta-analysis. J Hepatol 2020;73:523–532.
[3] Stockdale AJ, Chaponda M, Beloukas A, et al. Prevalence of hepatitis D virus infection in sub-Saharan Africa: a systematic review and meta- analysis. Lancet Glob Health 2017;5:e992–e1003.
[4] Rizzetto M, Hamid S, Negro F. The changing context of hepatitis D. J Hepatol 2021;74:1200–1211.
[5] Polaris Observatory Collaborators. Adjusted estimate of the prevalence of hepatitis delta virus in 25 countries and territories. J Hepatol 2024;80:232–242.
[6] Razavi HA, Buti M, Terrault NA, et al. Hepatitis D double reflex testing of all hepatitis B carriers in low-HBV- and high-HBV/HDV-prevalence countries. J Hepatol 2023;79:576–580.
[7] WHO. Guidelines for the diagnosis, care and treatment of persons with chronic hepatitis B infection. Geneva: World Health Organization; 2024. https://www.who.int/publications/i/item/9789240090903. [Accessed 19 October 2024].
[8] Global hepatitis report 2024: action for access in low- and middle-income countries. Geneva: World Health Organization; 2024. https://www.who.int/publications/i/item/9789240091672. [Accessed 9 April 2024].
[9] World Health Organization (WHO). Global health sector strategies on viral hepatitis, 2016-2021. Towards ending viral hepatitis. Geneva: WHO; 2016. https://www.who.int/publications/i/item/WHO-HIV-2016.06.
[10] Page MJ, McKenzie JE, Bossuyt PM, et al. The PRISMA 2020 statement: an updated guideline for reporting systematic reviews. BMJ 2021;372(71):1–10.
[11] Pandis N. Statistical inference with confidence intervals. Am J Orthod Dentofacial Orthop 2015;147:632–634.
[12] Andernach IE, Leiss LV, Tarnagda ZS, et al. Characterization of hepatitis delta virus in sub-Saharan Africa. J Clin Microbiol 2014;52(5):1629–1636.
[13] Pinho-Nascimento CA, Bratschi MW, Höfer R, et al. Transmission of hepatitis B and D viruses in an African rural community. mSystems 2018;3(5):e00120-18.
[14] Besombes C, Njouom R, Paireau J, et al. The epidemiology of hepatitis delta virus infection in Cameroon. Gut 2020;69(7):1294–1300.
[15] Noubissi-Jouegouo L, Atsama MA, Tagnouokam-Ngoupo PA, et al. Evolutionary trends in the prevalence of anti-HDV antibodies among patients positive for HBsAg referred to a national laboratory in Cameroon from 2012 to 2017. BMC Res Notes 2019;12(1):417.
[16] Butler EK, Rodgers MA, Coller KE, et al. High prevalence of hepatitis delta virus in Cameroon. Sci Rep 2018;8(1):11617.
[17] **Komas NP**, Ghosh S, Abdou-Chekaraou M, et al. Hepatitis B and hepatitis D virus infections in the Central African Republic, twenty-five years after a fulminant hepatitis outbreak, indicate continuing spread in asymptomatic young adults. PLoS Negl Trop Dis 2018;12(4):e0006377.
[18] Gomaa NI, Metwally LA, Nemr N, et al. Seroprevalence of HDV infection in HBsAg positive population in Ismailia, Egypt. Egypt J Immunol 2013;20(1):23–28.
[19] Tassachew Y, Belyhun Y, Abebe T, et al. Magnitude and genotype of hepatitis delta virus among chronic hepatitis B carriers with a spectrum of liver diseases in Ethiopia. Ann Hepatol 2023;28(1):1–6.
[20] Makuwa M, Mintsa-Ndong A, Souquière S, et al. Prevalence and molecular diversity of hepatitis B virus and hepatitis delta virus in urban and rural populations in northern Gabon in central Africa. J Clin Microbiol 2009;47(7):2265–2268.
[21] Groc S, Abbate JL, Le Gal F, et al. High prevalence and diversity of hepatitis B and hepatitis delta virus in Gabon. J Viral Hepat 2019;26(1):170–182.
[22] Ampah KA, Pinho-Nascimento CA, Kerber S, et al. Limited genetic diversity of hepatitis B virus in the general population of the offin river valley in Ghana. PLoS One 2016;11(6):e0156864.
[23] Sobajo OA, George UE, Osasona OG, et al. Seroprevalence, co-infection and risk of transmission of Hepatitis B and D virus among hospital attendees in two South-western states in Nigeria. J Immunoassay Immunochem 2023;44(1):133–146.
[24] **Lemoine M**, **Shimakawa Y**, **Njie R**, et al. PROLIFICA investigators, Acceptability and feasibility of a screen-and-treat programme for hepatitis B virus infection in the Gambia: the Prevention of Liver Fibrosis and Cancer in Africa (PROLIFICA) study. Lancet Glob Health 2016;4(8):559–567.
[25] Froeschl G, Hoelscher M, Maganga LH, et al. Hepatitis B, C and D virus prevalence in children and adults in Mbeya Region, Tanzania: results from a cohort study 2002 - 2009. Pan Afr Med J 2021;39:174.
[26] Djebbi A, Rebai WK, Bahri O, et al. Serological markers, viral RNA and genotype of hepatitis delta virus in HBs antigen positive Tunisian patients. Pathol Biol 2009;57:518–523.

[27] Mhalla S, Kadri Y, Alibi S, et al. Hepatitis D virus infection among hepatitis B surface antigen carriers and in "Isolated anti-HBc" antibodies profile in Central Tunisia. Hepat Mon 2016;16(1):e32354.
[28] Triki H, Said N, Ben Salah A, et al. Seroepidemiology of hepatitis B, C and delta viruses in Tunisia. Trans R Soc Trop Med Hyg 1997;91:11–14.
[29] Ouedraogo HG, Kouanda S, Grosso A, et al. Hepatitis B, C, and D virus and human T-cell leukemia virus types 1 and 2 infections and correlates among men who have sex with men in Ouagadougou, Burkina Faso. Virol J 2018;15(1):194.
[30] Belyhun Y, Liebert UG, Maier M. Clade homogeneity and low rate of delta virus despite hyperendemicity of hepatitis B virus in Ethiopia. Virol J 2017;14(1):176.
[31] Mansour W, Bollahi MA, Hamed CT, et al. Virological and epidemiological features of hepatitis delta infection among blood donors in Nouakchott, Mauritania. J Clin Virol 2012;55(1):121–126.
[32] Cunha L, Plouzeau C, Ingrand P, et al. Use of replacement blood donors to study the epidemiology of major blood-borne viruses in the general population of Maputo, Mozambique. J Med Virol 2007;79(12):1832–1840.
[33] De Paschale M, Ceriani C, Cerulli T, et al. Prevalence of HBV, HDV, HCV, and HIV infection during pregnancy in northern Benin. J Med Virol 2014;86(8):1281–1287.
[34] Ndzie Ondigui JL, Mafopa Goumkwa N, Lobe C, et al. Prevalence and risk factors of transmission of hepatitis delta virus in pregnant women in the Center Region of Cameroon. PLoS One 2024;19(6):e0287491.
[35] Makuwa M, Caron M, Souquière S, et al. Prevalence and genetic diversity of hepatitis B and delta viruses in pregnant women in Gabon: molecular evidence that hepatitis delta virus clade 8 originates from and is endemic in central Africa. J Clin Microbiol 2008;46(2):754–756.
[36] Andersson MI, Maponga TG, Ijaz S, et al. The epidemiology of hepatitis B virus infection in HIV-infected and HIV-uninfected pregnant women in the Western Cape, South Africa. Vaccine 2013;31(47):5579–5584.
[37] Torimiro JN, Nanfack A, Takang W, et al. Rates of HBV, HCV, HDV and HIV type 1among pregnant women and HIV type 1 drug resistance-associated mutations in breastfeeding women on antiretroviral therapy. BMC Pregnancy Childbirth 2018;18(1):504.
[38] Stockdale AJ, Chaponda M, Beloukas A, et al. Prevalence of hepatitis D virus infection in sub-Saharan Africa: a systematic review and meta-analysis. Lancet Glob Health 2017;5(10):e992–e1003.
[39] Hønge BL, Jespersen S, Medina C, et al., Bissau HIV cohort study group. Hepatitis B and Delta virus are prevalent but often subclinical co-infections among HIV infected patients in Guinea-Bissau, West Africa: a cross-sectional study. PLoS One 2014;10(9):e99971.
[40] Opaleye OO, Japhet OM, Adewumi OM, et al. Molecular epidemiology of hepatitis D virus circulating in Southwestern Nigeria. Virol J 2016;13:61.
[41] Wembulua BS, Le Gal F, Ndiaye O, et al. Hepatitis delta and liver disease among people living with hepatitis B with or without HIV Co-infection in Senegal. Liver Int 2025;45(3):e70026.
[42] Diop-Ndiaye H, Touré-Kane C, Etard JF, et al. Hepatitis B, C seroprevalence and delta viruses in HIV-1 Senegalese patients at HAART initiation (retrospective study). J Med Virol 2008;80(8):1332–1336.
[43] Winter A, Letang E, Vedastus Kalinjuma A, et al., KIULARCO Study Group. Absence of hepatitis delta infection in a large rural HIV cohort in Tanzania. Int J Infect Dis 2016;46:8–10.
[44] Katwesigye E, Seremba E, Semitala F, et al. Low sero-prevalence of hepatitis delta antibodies in HIV/hepatitis B co-infected patients attending an urban HIV clinic in Uganda. Afr Health Sci 2017;17(4):974–978.
[45] Souda S, Mwita JC, Cainelli F, et al. Seroprevalence and risk factors of hepatitis B, C and D virus infection amongst patients with features of hepatitis in a referral hospital in Botswana: a cross-sectional study. S Afr J Infect Dis 2021;36(1):275.
[46] Makhlouf NA, Morsy KH, Mahmoud AA. Hepatitis D virus infection among hepatitis B virus surface antigen positive individuals in Upper Egypt: prevalence and clinical features. J Infect Public Health 2019;12(3):350–356.
[47] Elzefzafy W, Soliman R, Saleh L, et al. Seroprevalence and epidemiological characteristics of HDV infection among HBV patients in the Nile Delta, Egypt. J Viral Hepat 2022;29(1):87–90.
[48] Aberra H, Gordien E, Desalegn H, et al. Hepatitis delta virus infection in a large cohort of chronic hepatitis B patients in Ethiopia. Liver Int 2018;38(6):1000–1009.
[49] Onyekwere CA, Audu RA, Duro-Emmanuel F, et al. Hepatitis D infection in Nigeria. Indian J Gastroenterol 2012;31:34–35.
[50] Alajab MB, Ibn Auf B, Rafei A, et al. Prevalence and clinical characterization of hepatitis D virus (HDV) infection among Sudanese patients with hepatitis B virus (HBV): a cross-sectional study. Ann Med Surg (Lond) 2024;86(9):5091–5095.
[51] Gourari S, Brichler S, Le Gal F, et al. Hepatitis B virus and hepatitis delta virus subtypes circulating in Algeria and seroprevalence of HDV infection. J Med Virol 2019;91:72–80.
[52] Luma HN, Eloumou SAFB, Okalla C, et al. Prevalence and characteristics of hepatitis delta virus infection in a tertiary hospital setting in Cameroon. J Clin Exp Hepatol 2017;7(4):334–339.
[53] Amougou MA, Noah DN, Moundipa PF, et al. A prominent role of Hepatitis D Virus in liver cancers documented in Central Africa. BMC Infect Dis 2016;16(1):647.
[54] Asmah RH, Boamah I, Afodzinu M, et al. Prevalence of hepatitis d infection in patients with hepatitis B virus-related liver diseases in Accra, Ghana. West Afr J Med 2014;33(1):32–36.
[55] Lunel-Fabiani F, Mansour W, Amar AO, et al. Impact of hepatitis B and delta virus co-infection on liver disease in Mauritania: a cross sectional study. J Infect 2013;67:448–457.
[56] Abdulkareem LO, Ndububa DA, Uhunmwangho AO, et al. Hepatitis D virus antibodies and liver function profile among patients with chronic hepatitis B infection in Abuja, Nigeria. J Infect Dev Ctries 2021;15(1):141–146.
[57] Nwokediuko SC, Ijeoma U. Seroprevalence of antibody to HDV in Nigerians with hepatitis B virus-related liver diseases. Niger J Clin Pract 2009 Dec;12(4):439–442.
[58] Olal SO, Akere A, Otegbayo JA, et al. Are patients with primary hepatocellular carcinoma infectious of hepatitis B, C and D viruses? Afr J Med Med Sci 2012;41(Supp l):187–191.
[59] Vray M, Debonne JM, Sire JM, et al. Molecular epidemiology of hepatitis B virus in Dakar, Sénégal. J Med Virol 2006;78:329–334.
[60] Yacoubi L, Brichler S, Mansour W, et al. Molecular epidemiology of hepatitis B and delta virus strains that spread in the mediterranean North East Coast of Tunisia. J Clin Virol 2015;72:126–132.
[61] European Association for the Study of the Liver. EASL 2017 Clinical Practice Guidelines on the management of hepatitis B virus infection. J Hepatol 2017;67:370–398.
[62] Sarin SK, Kumar M, Lau GK, et al. Asian-Pacific clinical practice guidelines on the management of hepatitis B: a 2015 update. Hepatol Int 2016;10:1–98.
[63] Terrault NA, Lok ASF, McMahon BJ, et al. Update on prevention, diagnosis, and treatment of chronic hepatitis B: AASLD 2018 hepatitis B guidance. Hepatology 2018;67:1560–1599.
[64] Palom A, Rando-Segura A, Vico J, et al. Implementation of anti-HDV reflex testing among HBsAg-positive individuals increases testing for hepatitis D. JHEP Rep 2022;4:100547.
[65] Brichler S, Trimoulet P, Roque-Afonso AM, et al. The diagnostic cascade for patients with hepatitis delta infection in France, 2018-2022: a cross-sectional study. Liver Int 2024;44:2858–2865.
[66] Lau DT, Jackson K, Picchio CA, et al. Availabilty of point of care HBV tests in resource-limited settings. Lancet Gastroenterol Hepatol 2024;9:1073–1076.
[67] **Lempp FA**, **Roggenbach I**, Nkongolo S, et al. A rapid point-of-care test for the serodiagnosis of hepatitis delta virus infection. Viruses 2021;13(12):2371.

Keywords: Hepatitis D; Epidemiology; Diagnosis; Survey.
Received 17 November 2024; received in revised form 12 June 2025; accepted 17 June 2025; Available online 3 July 2025

Journal of Hepatology, Volume *7*

Supplemental information

Hepatitis D epidemiology and access to diagnostic testing among healthcare providers in Africa: A multi-country survey

Maria Buti, C. Wendy Spearman, Karin Siebelt, and Manal El-Sayed

Hepatitis D epidemiology and access to diagnostic testing among healthcare providers in Africa: A multi-country survey

Maria Buti, C. Wendy Spearman, Karin Siebelt, Manal El-Sayed

Table of content

Table S1………………………………………………………………………………2

Table S1: The table shows the percentage of participants who were aware of HDV data on the following populations: Children, Hemodialysis patients and People Who Injects Drugs (PWIDs).

North Africa	Children	Dialysis	PWIDs
Algeria	NA	11%	NA
Egypt	NA	NA	NA
Libya	NA	NA	NA
Morocco	NA	NA	NA
Sudan	NA	NA	NA
Tunisia	NA	NA	NA
**West Africa**	**Children**	**Dialysis**	**PWIDs**
Benin	NA	NA	NA
Burkina Faso	6-20,5%	NA	4%
Côte d'Ivoire	NA	NA	NA
The Gambia	5%	2%	4%
Ghana	NA	NA	10%
Guinea	NA	NA	NA
Liberia	3%	NA	1%
Mali	NA	NA	NA
Mauritania	14%	32%	NA
Niger	NA	NA	NA
Nigeria	5,5-11,4%	0-58%	0,1-40%
Senegal	NA	NA	NA
Sierra Leone	NA	NA	NA
Togo	NA	NA	NA
**Central Africa**	**Children**	**Dialysis**	**PWIDs**
Cameroon	1-50,5%	2%	NA
Central African Republic	16%	NA	NA
Congo-Kinshasa	NA	NA	NA
Democratic Republic of Congo	NA	NA	NA
Gabon	50%	NA	NA
**East Africa**	**Children**	**Dialysis**	**PWIDs**
Burundi	NA	15%	NA
Ethiopia	NA	NA	NA
Kenya	13%	24.3%	NA
Malawi	1,5-2%	NA	NA
Mauritius	NA	NA	NA
Mozambique	NA	NA	NA
Rwanda	NA	NA	NA
Somalia	NA	NA	NA
South Sudan	NA	NA	NA
Uganda	4-5%	1,5-45%	5-50%
Tanzania	3,3-60%	14%	20-27%
Zambia	1-5%	7%	10%
Zimbabwe	NA	NA	NA
**Southern Africa**	**Children**	**Dialysis**	**PWIDs**

**Botswana**	NA	NA	NA
**Eswatini**	NA	10%	NA
**Namibia**	NA	NA	NA
**Lesotho**	NA	NA	NA
**Namibia**	NA	NA	NA
**South Africa**	NA	NA	NA
